# Supplementary material for: The effect of cue length and position on noticing and learning of determiner agreement pairings: Evidence from a cue-balanced artificial vocabulary learning task
Source: PLoS One. 2024 Jul 23;19(7):e0302355. doi: 10.1371/journal.pone.0302355 (PMC11265659; doi:10.1371/journal.pone.0302355)
Supplement: S1 Table — (DOCX) [file pone.0302355.s001.docx]

**S1. Determiner and noun item list**

| Determiner | Known (trained) Noun | Translation | Determiner | Novel (untrained) Noun |
| --- | --- | --- | --- | --- |
| mo | grupswek | the raspberry | mo | grupdang |
| mo | kemypo | the table | mo | grupello |
| zi | megleshre | the lamp | mo | grupasa |
| lu | vref | the shirt | mo | grupopo |
| zi | arotep | the salmon | mo | gruptika |
| mo | grupble | the flower | lu | esulata |
| zi | astaga | the train | lu | masulin |
| zi | pipshre | the broccoli | lu | versultik |
| zi | atoree | the pot | lu | porsulap |
| lu | resulm | the ship | lu | chesulwer |
| zi | butoshre | the deer | zi | indoshre |
| zi | alir | the pen | zi | eboushre |
| mo | gusulter | the chipmunks | zi | huelcshre |
| mo | neyn | the bus | zi | boshre |
| mo | grupaug | the chair | zi | etalshre |
| lu | tesulni | the apple | zi | altinns |
| zi | vandashre | the plane | zi | agranti |
| mo | gruperv | the zebra | zi | awantoo |
| zi | asei | the cake | zi | amuke |
| mo | danydit | the frog | zi | andasaga |
| lu | nandef | the bacon | mo | binysap |
| mo | mayb | the burger | mo | sakyla |
| lu | pasulpei | the trash can | mo | foyboer |
| lu | womf | the eraser | mo | beytr |
| lu | kembof | the bench | mo | gopybe |
| mo | gruprit | the car | lu | keimf |
| lu | nisult | the bike | lu | sembof |
| zi | slushre | the marker | lu | bfelf |
| lu | jiklaf | the shark | lu | bokrof |
| mo | guyter | the couch | lu | tekif |

List of determiner and noun pairs used for the study. There are six possible combinations of place (beginning, middle, end of word) and length (long or short). For each possible combination, there are five trained (known) and five novel (untrained) words. There are three determiners matched to two of the possible cue combinations (*lu* with *sul* (middle, long) and *f* (end, short), *zi* with *shre* (end, long) and *a* (beginning, short), and *mo* with *grup* (beginning, long) and *y* (middle, short).
