# Supplementary material for: The effect of cue length and position on noticing and learning of determiner agreement pairings: Evidence from a cue-balanced artificial vocabulary learning task
Source: PLoS One. 2024 Jul 23;19(7):e0302355. doi: 10.1371/journal.pone.0302355 (PMC11265659; doi:10.1371/journal.pone.0302355)
Supplement: Attachment — Submitted filename: Response to Reviewers.docx [file pone.0302355.s006.docx]

Response to Reviewers:

On my first submission, I entered the wrong time frame in which participants for this study were recruited and took part in the experiment. All of my participants for this study took part after IRB approval was granted. I have attached pdfs from the online platform that we used, Gorilla.sc, with the date stamps for all 54 participants in the study that show dates all after the indicated IRB approval date.
